# Dual use of Medicare and the Veterans Health Administration: are there adverse health outcomes?

**DOI:** 10.1186/1472-6963-6-131

**Published:** 2006-10-09

**Authors:** Fredric D Wolinsky, Thomas R Miller, Hyonggin An, Paul R Brezinski, Thomas E Vaughn, Gary E Rosenthal

**Affiliations:** 1 Center for Research in the Implementation of Innovative Strategies in Practice (CRIISP), Iowa City Health Care System, 601 Highway 6 West, Iowa City, IA52246, USA; 2 Health Management and Policy, College of Public Health, the University of Iowa, 200 Hawkins Drive, Iowa City, IA52242, USA; 3 Internal Medicine, Carver College of Medicine, the University of Iowa, 200 Hawkins Drive, Iowa City, IA52242, USA; 4 Biostatistics, College of Public Health, the University of Iowa, 200 Hawkins Drive, Iowa City, IA52242, USA; 5 United States Air Force, USA

## Abstract

**Background:**

Millions of veterans are eligible to use the Veterans Health Administration (VHA) and Medicare because of their military service and age. This article examines whether an indirect measure of dual use based on inpatient services is associated with increased mortality risk.

**Methods:**

Data on 1,566 self-responding men (weighted N = 1,522) from the Survey of Assets and Health Dynamics among the Oldest Old (AHEAD) were linked to Medicare claims and the National Death Index. Dual use was indirectly indicated when the self-reported number of hospital episodes in the 12 months prior to baseline was greater than that observed in the Medicare claims. The independent association of dual use with mortality was estimated using proportional hazards regression.

**Results:**

96 (11%) of the veterans were classified as dual users. 766 men (50.3%) had died by December 31, 2002, including 64.9% of the dual users and 49.3% of all others, for an attributable mortality risk of 15.6% (p < .003). Adjusting for demographics, socioeconomics, comorbidity, hospitalization status, and selection bias at baseline, as well as subsequent hospitalization for ambulatory care sensitive conditions, the independent effect of dual use was a 56.1% increased relative risk of mortality (AHR = 1.561; p = .009).

**Conclusion:**

An indirect measure of veterans' dual use of the VHA and Medicare systems, based on inpatient services, was associated with an increased risk of death. Further examination of dual use, especially in the outpatient setting, is needed, because dual inpatient and dual outpatient use may be different phenomena.

## Background

There are 9.5 million US veterans aged 65 years old or older [1] who are eligible to use the Veterans Health Administration (VHA) system due to their military service, and to use and have their care in the private health care delivery system paid for by Medicare due to their age [1-6]. The implications of such dual use can be both positive and negative [7-10]. On the positive side, dual use provides veterans with access to more sources and sites of health care and to a greater diversity of health care product lines [4-6]. Those services, however, are received from multiple health professionals in two distinct and disarticulated delivery systems. Thus, on the negative side, dual use may decrease the likelihood that veterans receive continuously coordinated care [3,10,11].

When older adults with multiple chronic conditions receive services from several different providers who are not centrally managed and coordinated, monitoring effectiveness decreases, and the likelihood of medical errors and contraindicated and competing regimens increases [12]. Indeed, the absence of "a continuous (and coordinating) healing relationship" [13] increases the risk of hospitalization for ambulatory care sensitive conditions (ACSCs). Based on Rutstein et al.'s [14-16] early studies of preventable hospitalization and enhanced by second generation studies during the 1990 s, [17-20] hospitalizations for ACSCs were recently formalized as the most appropriate and policy relevant community markers of health care quality by the Agency for Healthcare Research and Quality (AHRQ) [21,22]. The underlying assumption is that if quality care is received, attendant efforts at comprehensive care management and primary and secondary prevention can eliminate or at least delay the need for such hospital episodes. Ultimately, the lack of continuity of care and hospitalization for ACSCs are thought to increase the risk of mortality.

It is not clear how many older veterans use both of their health care entitlements. One GAO report indicated that among Medicare-eligible veterans who used any health care services in 1990, 81% used Medicare only, 9% used only the VHA, and 10% used both systems [23]. In contrast, Fisher and Welch reported that 52% of all VHA patients who were Medicare eligible filed at least one Medicare benefit claim within a single year, [2] and another GAO report suggested that 54% of Medicare-eligible veterans were dual users [24]. VIReC recently concluded that although 90% of older VHA patients were enrolled in Medicare, 22% used only VHA services, 30% used only Medicare services, and 43% used services from both sources [25]. Thus, dual use estimates range from 10% to 68%. This wide range of dual use estimates is understandable, and results from differences in sample selection and design. The lowest estimated dual use rate comes from the only population-based study, which includes veterans who use few, if any, VHA health services. In contrast, the higher dual use rates are from samples of veterans who were current users of the VHA.

Like the prevalence of dual use, little is known about its antecedents. Among the few extant studies, Agha et al. found that veterans who primarily use VHA facilities had lower education, income, and health status [26]. Distance to the nearest VHA facility has also been reported to be predictive of dual use (an inverse relationship) [7,9,27,28]. None of these studies, however, was comprehensive in its consideration of potential precursors of dual use, longitudinal by design, or involved a representative sample of veterans. Thus, a considerable knowledge gap exists with regard to the potential adverse effects of dual use among veterans.

In this article, the potential adversity of dual use among older male veterans is examined using an innovative, secondary analysis of a comprehensive and publicly available data set. The hypothesis is that dual use based on inpatient services among older male veterans ultimately increases their risk of mortality. It is assumed that the etiological mechanism resides in the lack of continuously coordinated health care, [12] and its effect on a cascade of subsequent adverse outcomes including, but not limited to increasing the risk of hospitalization for ACSCs. As *Crossing the Quality Chasm *makes clear, a continuously coordinated health program that spans disparate delivery systems is essential to providing quality health care and avoiding premature death [13].

## Methods

### The AHEAD data set

To evaluate whether dual use based on inpatient services increases the risk of mortality, data are taken from the Survey on Assets and Health Dynamics among the Oldest Old (AHEAD). Because over-sampling was used to increase the number of African Americans, Hispanics, or Floridians in the AHEAD, the data are weighted to adjust for the unequal probabilities of selection due either to the multi-stage cluster sampling design and/or the over-sampling. The AHEAD data set was selected for three reasons. First, it is a nationally representative probability sample that includes 2,911 men and 4,536 women who were 70 years old or older at their baseline interviews in 1993–94. Because only 57 women (1%) were veterans, these analyses are restricted to men. Among the men, 1,574 (54%) are veterans. This provides a large, nationally representative sample, evenly distributed between veteran and non-veteran men. Second, the AHEAD baseline survey data have been linked to Medicare claims from January 1989 through December 1996. Third, the AHEAD survey and Medicare claims data have also been linked to the National Death Index (NDI) through December 2002. This provides a nine-year window for examining the association of an indirect dual use measure based on inpatient services with mortality, during which 52% (1,524) of these men died.

### The dual use measure

Unfortunately, the AHEAD is not linked to VHA claims. Thus, an indirect measure of dual use based on inpatient services was constructed. This was done by building on the extant literature addressing differences between self-reports and administrative records of health services use. It has been well established that concordance between the two is not perfect, and that the discordance is not easily predicted [29-37]. The magnitude of the discordance is primarily influenced inversely by the salience of the event, and directly by the length of the recall period [33-35]. Hospital episodes are considered to be the most salient events, and for them a 12-month window provides a reasonable balance between recall abilities and the incidence of hospitalization [29,37]. Discordance has also been shown to be positively associated with the number of events during the reporting period, and to a lesser extent with demographic, social, and health factors, although these associations have not been consistently observed [37]. Elsewhere, we have shown that in the AHEAD, the concordance of self-reports and Medicare claims is high for both any (vs. none; **κ **= .763) and the precise number of (**κ **= .663) hospital episodes over a 12-month window [38].

Building on this literature, the indirect measure of dual use based on inpatient services was constructed as follows. At baseline, each AHEAD man was asked whether he was hospitalized overnight during the previous 12 months, and if he had been, how many times this occurred. Using each AHEAD man's baseline interview date, corresponding data were harvested from his Medicare claims. The self-reports were then compared to the claims results. If the AHEAD man reported at least one more hospital episode than was found in his Medicare claims he was considered an over-reporter.

Although straightforward, this approach has four limitations that, although addressable, warrant further mention. First, this approach ignores any dual use that occurs solely in the outpatient setting. As indicated above, however, the veridicality of such measures is substantially less than that obtained by focusing just on the inpatient setting [29-37]. Indeed, in the AHEAD, we have shown that the concordance of self-reports and Medicare claims is low for both any (vs. none; **κ **= .248) and the precise number of (**κ **= .347) physician visits over a 12-month window [38]. Second, this approach ignores the extent of the over-reporting. In these data, however, 79% of those who self-reported more hospital episodes than were found in their Medicare claims over-reported by only one hospital episode. Third, this approach may confound dual use with the proclivity for hospitalization. That is, by definition, all dual users have reported that they were hospitalized. This confound, however, can be addressed by including in the analysis a binary marker for whether the AHEAD man reported being hospitalized. Fourth, this approach does not actually identify dual use, because dual use can only occur among veterans.

Isolation of the dual use effect, however, can be readily achieved by constructing a set of four dummy variables that reflects the cross-classification of over-reporting with veteran status. The analyses reported here include three of these markers – veterans who over-reported (and thus are considered to be dual users of VHA and Medicare), veterans who accurately reported, and non-veterans who over-reported (but could not be dual users of the VHA and Medicare). The omitted or reference group is that of non-veterans who accurately reported their number of hospital episodes.

In this approach, the effect of the dummy variable for veterans who over-reported their number of hospital episodes accurately reflects the mortality risk of dual use of VHA and Medicare based on inpatient services. The hypothesis is that this effect will be statistically significantly greater than unity, reflecting the increased mortality risk associated with dual use of VHA and Medicare. It is further hypothesized that the effect of the dummy variable for non-veterans who over-reported will not be statistically significantly different than unity, because non-veterans have no access to VHA.

### Mortality

Vital status was obtained by linking the AHEAD to the NDI. The NDI files indicate whether each AHEAD man died, and if so, provide the month and year of death. Also provided, but not used in these analyses, are indicators of the probability of the match using standard criteria [39] developed by the National Center for Health Statistics (NCHS), and detailed ICD9-CM codes for the cause of death.

### Selection bias

Of the 2,911 AHEAD men, the survey data was provided by a proxy-respondent (usually the spouse) for 391 (13%). Because the literature [29-38] on reporting discrepancies assumes self-respondents, analyses were restricted to the 2,520 AHEAD men who were self-respondents. Linkage to the Medicare claims was not available for an additional 954 AHEAD men (38%). Of these, 182 refused to consent to having their Medicare claims accessed, and for the remainder (772 men) sufficiently accurate information to facilitate the linkage process was not available. All 954 AHEAD men whose self-reported survey data could not be linked to their Medicare claims were excluded from the analyses. Thus, of the 2,911 AHEAD men, the analyses reported here were restricted to the 1,566 (54%; weighted N = 1,522) who were self-respondents and whose survey data was linked to their Medicare claims.

The exclusion of so many AHEAD men from these analyses raised the potential for selection bias. This potential for selection bias was addressed using propensity score methods. Developed by Rubin, [40] popularized by Rosenbaum and Rubin, [41] and illustrated by D'Agostino, [42] propensity scores are traditionally obtained by using multiple logistic regression to model a binary outcome reflecting group assignment in observational (vs. randomized controlled trial) studies. Here, propensity scores were used to model selection bias. To obtain the selection bias propensity scores, a multivariable logistic regression was conducted using all appropriate baseline covariates, including veterans' status, to predict exclusion of self-respondents from the analytic sample. The fit of the propensity score model was reasonably robust (C-statistic [43] = .638), and there was no evidence of heteroscedastic error (Hosmer-Lemeshow statistic [44] p value = .934). Thus, adding the obtained predicted probabilities of exclusion to the final analytic model among the restricted sample should be an appropriate adjustment for potential selection bias (at least in terms of the data available for analysis). This propensity score regression approach, however, assumed additive linearity in the relationships of interest. Therefore, as an added safeguard, the results were replicated using the more popular stratification approach. In this approach, the final model (excluding the propensity score) was re-estimated separately within strata based on the propensity score. If the results were robust across these strata, greater confidence could be had in the selection bias adjustment process.

### Exploring the etiological mechanism

Although the main focus of this article was whether dual use of the VHA and Medicare systems, indirectly indexed by veterans' over-reporting their number of hospital episodes, was associated with mortality, a secondary interest involved the etiological mechanism through which this association occurred. It was assumed that (a) dual use decreased the likelihood of receiving continuously coordinated health care, (b) the lack of continuously coordinated health care increased the risk of subsequent hospitalizations for ACSCs, and (c) these three factors (dual use, the lack of continuously coordinated care, and hospitalization for ACSCs) increased the risk of mortality. Left unspecified was whether the effect of dual use was direct, indirect (through its intermediary [e.g., falling domino] effects on the lack of continuously coordinated care and the increased risk of subsequent hospitalization for ACSCs), or a combination of the two. To begin exploring these issues, a final adjustment in the modeling process was made for subsequent hospitalizations for ACSCs. This was done by creating a binary marker indicating whether the subject had one or more hospitalizations for ACSCs after their baseline interview but before January 1, 1997. This marker was coded one for subjects with one or more such hospitalizations based on AHRQ's computerized criteria, [21,22] and zero otherwise.

Although a measure of continuity of care based on the Medicare Part B (outpatient) claims has been developed for use in these data, [45] it could not be incorporated into these analyses. The reason was that this measure would have yielded biased estimates of continuity of care among dual users because it would have ignored their VHA outpatient visits, as those data were not available. Not being able to adjust for continuity of care severely constrained the ability to further explore the etiologic mechanism through which dual use was associated with mortality.

### Covariates

In addition to adjusting for potential selection bias, fifteen covariates were included to ensure that the estimated association of dual use with mortality was fully independent from other background factors. These covariates included age, race, education, income, assets, activities of daily living (ADLs), instrumental ADLs (IADLs), self-rated health, five chronic diseases, cognitive ability, and depressive symptoms. Age was measured in years. Race was measured by a set of three dummy variables for Hispanics, African Americans, and other non-Caucasians (with Caucasians as the reference group). Education was measured by a dummy variable contrasting high school graduates (and above) with those having less education. Income was measured by a binary marker for having less than $15,000 in annual income. Household wealth was measured as the sum of all reported assets net of debt, and was coded by a binary marker for having $19,000 or less in total wealth. ADLs were measured by a count of the number of five items (e.g., bathing) that the subject reported having any difficulty performing. Similarly, IADLs were measured by a count of the number of five items (e.g., meal preparation) that the subject reported having any difficulty performing. Self-rated health was measured by a set of four dummy variables for excellent, very good, fair, or poor responses to the standard question asking subjects to rate their health (with a good response as the reference group). Five binary variables (1 = yes, 0 = no) were used to indicate whether the subject reported having ever been told by a physician that he had cancer, diabetes, heart disease, lung disease, or a stroke. Cognitive ability was measured using the 7-item version of the Telephone Index of Cognitive Status, which ranged from 0 (worst) to 15 (best) (TICS-7) [46]. Depressive symptoms were measured as the number of symptoms endorsed using an 8-item version of the CES-D [47].

### Analytic approach

Because the month and year of death are known, proportional hazards models were the appropriate statistical approach for estimating the effect of dual use on mortality [48]. A series of proportional hazards models were estimated that initially assessed the crude effect of the set of three dummy variables reflecting the cross-classification of veteran status with over-reporting, and then serially decomposed that effect. The decomposition approach involved four stages that serially introduced (a) the binary marker for reporting any hospital episodes in the year prior to baseline, (b) the binary marker for whether post-baseline hospitalizations for ACSCs occurred, (c) the fifteen covariates, and finally, (d) the propensity score adjustment for potential selection bias. Sensitivity analyses were then conducted in which the final model, excluding the propensity score adjustment for selection bias, was re-estimated within each propensity score strata.

### Institutional review

Because the research reported here involved the linkage of public use data files containing the AHEAD survey data with restricted data from the NDI files and Medicare claims, three layers of institutional review and approval were obtained. The first involved review and approval of the research and restricted data protection plans associated with the main NIH grant (R01 AG022913) by the AHEAD's Data Confidentiality Committee (DCC). These were approved by the AHEAD DCC on February 20, 2003 (#2003–006). The second layer of review and approval involved the University of Iowa Institutional Review Board (UI-IRB). The UI-IRB approved the original protocol on March 24, 2003, and has subsequently approved the protocol at all annual reviews (including appropriate modifications to incorporate the second NIH grant – R03 AG027741 – which specifically focused on dual use). The third layer of review and approval involved the Centers for Medicare and Medicaid Services (CMS). CMS approved the Data Use Agreement (DUA 14807) to access the Medicare claims for this research on March 3, 2005.

## Results

### Descriptive

The analytic sample consists of the 1,566 AHEAD men (weighted N = 1,522) who were self-respondents and whose survey data were linked to their Medicare claims. Table 1 shows the means or percentages for the variables in the final model separately for each of the four distinct groups based on the cross-classification of over-reporting and veteran status. Overall, the mean age of the AHEAD men was 77 years (SD = 6), 7% were Hispanic, 11% were African American, 1% were of another race other than Caucasian, and 82% were Caucasian. Forty-five percent had not graduated from high school, 40% reported $15,000 or less in household income, and 25% indicated that they had less than $19,000 in wealth. The mean number of ADLs was 0.3 (SD = 0.7; 86% had none), and the mean number of IADLs was 0.4 (SD = 0.9; 78% had none). Twelve percent reported their health as excellent, 22% as very good, 32% as good, 22% as fair, and 11% as poor. Cancer was reported by 14%, diabetes was reported by 12%, heart disease was reported by 33%, lung disease was reported by 11%, and stroke was reported by 10%. The mean cognitive status score was 12 (SD = 3; 24% had the maximum score), and the mean number of depressive symptoms was 1.4 (SD = 1.9; 46% had none). Being hospitalized in the year prior to baseline was reported by 23%. Slightly more than half (55%) were veterans. Fourteen percent (226) experienced one or more post-baseline hospitalizations for ACSCs. By December 31, 2002, 817 (52%) of the AHEAD men had died.

Comparisons across columns (within rows) in Table 1 indicated that veterans had significant mortality risk advantages over non-veterans in terms of age, majority status, education, income, wealth, self-rated health, ADLs, IADLs, cognitive status, depressive symptoms, and subsequent hospitalization for ACSCs. In contrast, veterans had significant mortality risk disadvantages compared to non-veterans in terms of cancer and diabetes. Comparisons within veterans or within non-veterans indicated that those who over-reported their number of hospital episodes had considerably greater mortality risk due to their greater morbidity levels, regardless of veteran status.

### Over-reporting

As indicated in Table 1, the number of self-reported hospital episodes exceeded that found in their linked Medicare claims for 96 (10.8%) of the veterans vs. 60 (9.5%) of the non-veterans. The minimal difference between these crude over-reporting rates (odds ratio = 1.217; p = .261), however, is misleading. Adjusting for age, race, and self-rated health status using multivariable logistic regression revealed that veterans were 50% more likely than non-veterans to over-report (adjusted odds ratio = 1.496; p = .046). Moreover, veterans who over-reported their hospital episodes had the same number of physician visits in their Medicare claims as veterans who did not (79.9% of veterans who over-reported had physician visits vs. 80.9% of veterans who did not; both medians = 5). In contrast, non-veterans who over-reported their hospital episodes had substantially more physician visits in their Medicare claims than non-veterans who did not (87.7% of non-veterans who over-reported had physician visits vs. 84.6% of non-veterans who did not; medians = 8 and 5, respectively). Finally, among those who reported hospital episodes, 48.4% of veterans over-reported the number vs. 41.8% of non-veterans.

### Proportional hazards regression

Table 2 contains the adjusted hazards ratios (AHRs) obtained from the final proportional hazards regression model. Note that these AHRs were adjusted for the binary marker for reporting any hospital episodes in the year prior to baseline, the binary marker for whether post-baseline hospitalizations for ACSCs occurred, the fifteen covariates, and the propensity score adjustment for potential selection bias. These results support both study hypotheses. That is, compared to non-veterans who accurately reported their number of hospital episodes, veterans who over-reported their number of hospital episodes (i.e., the indirect measure of dual use of VHA and Medicare, based on inpatient services) had an increased relative risk of dying (AHR = 1.561; p = .009). But, non-veterans who over-reported their number of hospital episodes did not have an increased relative risk of dying (AHR = 0.775; p = .191) compared to non-veterans who accurately reported their number of hospital episodes. Similarly, veterans who accurately reported their number of hospital episodes did not have an increased relative risk of dying (AHR = 1.168; p = .079) compared to non-veterans who accurately reported their number of hospital episodes.

The effects of the covariates on mortality in the final model were as expected. The relative risk of mortality significantly increased with age (AHR = 1.099 per year; p < .001), IADLs (AHR = 1.110 for each; p = .019), having poor vs. good self-rated health (AHR = 1.574; p < .001), and having diabetes (AHR = 1.311; p = .015), heart disease (AHR = 1.197; p = .025), lung disease (AHR = 1.458; p < .001), or a history of stroke (AHR = 1.439; p < .001). The relative risk of mortality was significantly reduced (AHR = 0.752; p = .011) among those having very good vs. good self-rated health, and among those having better cognitive status (AHR = 0.945 for each point of improvement on the TICS-7; p < .001). As expected, men with subsequent hospitalizations for ACSCs had increased risk of dying (AHR = 1.874; p < .001). The propensity score adjustment, however, was not associated with mortality (AHR = 0.893; p = .795).

### Sensitivity analysis

Because the propensity score regression adjustment approach assumed additive linearity in the relationships of interest, sensitivity analyses were conducted. This involved using the propensity score stratification approach, in which the final model (excluding the propensity score) was re-estimated separately within strata based on the propensity score. Although quintiles are used most often [40-42], tertiles were used here to balance the distribution of dual users across strata. Those analyses yielded remarkably consistent AHRs (1.465, 1.515, and 1.531 for the lowest to highest propensity score tertiles), indicating that the results shown in Table 2 are not artifacts of the selection bias propensity score adjustment approach.

## Discussion

This article evaluated the association between an indirect measure of inpatient-based dual use of the Medicare and VHA systems by older male veterans using a large, nationally representative sample. Among the 864 veterans with linked data, 96 or 11% were classified as dual users. This dual use rate is remarkably consistent with the 10% estimate reported in the only other population-based study of dual use among veterans, [23] and is substantially lower than estimates obtained from studies limited to veterans who use the VHA system [2,24,25]. By December 31, 2002 there had been 766 deaths among the AHEAD men (50.3%), including 64.9% of the dual users and 49.3% of all others, for an attributable mortality risk of 15.6% (p < .003). After adjusting for prior hospitalization, fifteen covariates, post-baseline hospitalization for ACSCs, and selection bias, male veterans who were dual users (based on inpatient services) had a 56.1% greater relative risk (p = .009) of mortality than non-veterans who accurately reported their number of hospital episodes.

The presumed etiological mechanism that accounts for this observed association is this: (a) dual use increases the risk of uncoordinated and poorly managed care; (b) the risk of uncoordinated and poorly managed care increases the risk of being hospitalized for ACSCs (and other intermediary problems); and, (c) taken together, these factors ultimately lead to the increased risk of mortality. Although subsequent hospitalization for ACSCs substantially increased the risk of mortality (AHR = 1.874; p < .001), adjusting for it did not appreciably alter the risk of mortality associated with dual use. In the absence of a marker for the continuity of care in these data, the precise magnitude of the direct effect of dual use based on inpatient services on mortality remains unknown.

Continuity of care has been conceptualized several different ways, including interpersonal [12,49] (or relational) continuity, informational continuity, [12] and site/team (or management) continuity [50]. The common theme is that continuity of care has positive effects on health outcomes because it (a) builds the practitioner's tacit knowledge of the patient, and (b) enhances trust between the patient and practitioner. In their exhaustive review of the literature, Saultz and Lochner [51] found that interpersonal continuity was associated with improved delivery of preventive services and lower hospitalization rates. More specifically, Gill and Mainous have shown that interpersonal continuity significantly reduces the risk of hospitalization for ACSCs and for emergency department visits [52-54]. Ettner has shown that having a usual physician decreases the likelihood of engaging in substance abuse and increases the probability of having annual preventive medical visits [55]. Although none of these studies involved veterans, Wasson and colleagues reported that continuity of care among male veterans 55 years old or older who used the VHA was associated with decreased use of the emergency department, shorter lengths of stay, and less time spent in ICUs [56].

Several important issues, however, have yet to be addressed in the literature evaluating the association between continuity of care and improved health outcomes. These include the need to separate the effects of continuity of care from access to care, [17,57,58] whether access to a single provider is preferable to an ongoing relationship with a practice site, [59] and the threshold at which the continuity of the patient-practitioner relationship manifests an effect [60]. Furthermore, little is known about whether and how the effect of continuity of care varies by patient (e.g., general health, specific situations, preferred involvement in care decisions) and/or by provider characteristics (e.g., specialty, interpersonal skills, workload).

Despite its strengths, this study has several important limitations. The more salient of these are that: (a) condition-specific analyses could not be performed; (b) provider specialty-mix was not known; (c) VHA claims (both inpatient and outpatient) were unavailable, and Medicare outpatient claims were not used; (d) information on local system characteristics and organizational factors was lacking; (e) dual use was treated solely as a static (baseline) factor; (f) continuity of care was unmeasured; and, (g) unobserved confounders remain potential threats to the internal validity of this observational study. Further research is needed to address these concerns.

The last concern, the potential for unobserved confounders, is always present in observational research. Particularly important here, however, is whether the veterans who over-reported their number of hospital episodes had more access to non-Medicare health insurance coverage than the non-veterans who over-reported. This would be especially important if such access resulted in no claims for a given hospital episode being submitted to Medicare, which would account for the greater adjusted likelihood of veterans vs. non-veterans to over-report their number of hospital episodes (relative to their Medicare claims).

Section 1862(b) of the Social Security Act {42 USC Section 1395y(b)(5)} indicates that if the older adult has non-Medicare insurance from certain sources (primarily from employer-based group health plans from active or spousal-active employment, or from retained work-based health benefits after retirement; but also due to end-stage renal disease, black lung, workers compensation, or no-fault accident entitlements), Medicare becomes the secondary payer. It is likely, however, that the majority of older adults in our analytic sample who had private insurance, had it to cover the gaps in Medicare, because it would make no sense for them to pay for private insurance to cover inpatient services that Medicare routinely covers. This is especially the case in light of the fact that all of the older adults in our analytic sample have Medicare Part A. For patients like these, a claim would still be submitted to Medicare for most of the elements (charges) associated with the hospital episode. And as a result, having such additional non-Medicare coverage would have no bearing on our findings, because the hospital episodes in question would still be documented in the patients' Medicare claims. Unfortunately, the insurance data available in the AHEAD study are not sufficiently granular to determine with certainty whether non-Medicare insurance coverage is principally Medi-gap coverage.

We were, however, able to examine the prevalence of employment-based insurance and that of several types of self-reported non-Medicare health insurance coverage among veteran and non-veteran over-reporters. Although data on employment-based insurance were not collected at baseline, they were obtained at the first (1995) follow-up interviews. In those data there was no difference (chi-squared test for cross-classifications; p = .611) in rates of having employment-based insurance between veterans and non-veterans who over-reported their hospital episodes.

In terms of other non-Medicare insurance coverage, there were no significant differences at baseline (1993) in terms of working for pay (p = .364), having long-term care insurance (p = .156), the number of health insurance policies (p = .106), having only Medicare Part A (p = .715), having Medicare Part A and Medicaid (p = .194), having Medicare Part A and some other insurance (p = .642), and having only Medicare Parts A and B (p = .231). Indeed, the only significant difference observed (p = .012) was that 80% of veterans who over-reported had Medicare Parts A and B and some other health insurance, compared to 62% of non-veterans who over-reported. That difference, however, is not sufficient to explain away our findings, because it represents only 17 veterans who over-reported. Moreover, this is precisely the group who would be most likely to have purchased non-Medicare insurance to cover the gaps in Medicare coverage. Thus, we did not find any plausible evidence of this particular form of unobserved confounding.

Accordingly, we believe that these results are sufficiently robust to have important policy implications – the indirect measure of dual use based on inpatient services was significantly associated with increased mortality, and the likely etiological mechanism involves poor coordination between VHA and Medicare. How can coordination between the VHA and Medicare be improved? The minimum requirement for better coordination is information access, particularly access to the medical record [12,45,51]. In this regard, the VHA has a substantial advantage – its electronic medical record system can already be shared from one VHA medical center or community based outpatient center to another and from one provider to another. Indeed, the VHA's electronic health record received the 2006 "Innovations in American Government Award" from Harvard University because not a single medical record of any veteran residing in the US Gulf Coast area was lost during the catastrophic 2005 hurricane season. And, the medical records for every one of the veterans in that area who were evacuated and subsequently relocated were immediately available to the VHA facilities and providers that took over their treatment and care management.

Unfortunately, the same can not be said for Medicare, or for the private health care delivery systems that rely on it for their reimbursement. Despite longstanding calls by the IOM and others [13] to have inpatient and outpatient electronic medical records universally in place, as well as a recent but unfunded mandate to achieve this from US President GW Bush, it has not happened and is not likely to happen anytime soon [61]. Moreover, although most non-VHA hospitals already have an electronic medical record system, those systems are limited because they typically only contain information generated locally (i.e., at that particular facility). Furthermore, given the substantial variations in computer and software systems, even hospitals in the same service area are not able to readily share information.

The development of local health information infrastructures is a promising development that may help to overcome these limitations, and the Indiana Network for Patient Care (INPC) is an early exemplar of this approach. INPC shares data from five hospital systems that include 15 separate hospitals, county and state public health department records, Indiana Medicaid, and pharmaceutical cooperatives [62]. Cross-institutional access to this information via INPC or other local health information infrastructures is the first step in inter-system coordination. Although advocates suggest considerable potential for health information infrastructures to transform health care by improving quality and lowering costs, [63] cynics counter that the history of health information technology has been mostly "hope and hype" with little to show at the moment for the substantial up-front investments that were required [64]. Nonetheless, facilitating the encouragement and support of these initiatives appears warranted. Moreover, because the VHA's electronic medical record system mimics several of those already involved in health information infrastructures like the INPC, the basic building blocks for the rapid development of a national infrastructure may already exist.

## Conclusion

Male veterans who were dual users of both VHA and Medicare, based on over-reporting their number of hospital episodes compared to data from their Medicare claims, had a 56.1% greater relative risk (p = .009) of mortality than non-veterans who accurately reported their number of hospital episodes. This relationship was quite robust, despite adjustment for hospitalization previous to the study period, numerous potential confounders, post-baseline hospitalization for ACSCs, and selection bias. The presumed etiological mechanism is that dual use increases the risk of uncoordinated and poorly managed care, which is especially important in the treatment and management of older adults with multiple chronic conditions.

## Competing interests

The author(s) declare that they have no competing interests.

## Authors' contributions

FDW conceived of the study, wrote both grant applications, designed the analyses, interpreted the results, and drafted and revised the manuscript. TRM cleaned and linked all of the data files, and conducted all of the statistical analyses. HA assisted in the design and oversight of the statistical analyses and their interpretation. PRB reviewed and synthesized the literature on continuity of care, and reviewed appropriate Medicare reimbursement regulations. TEV reviewed and synthesized the literature on dual use among older veterans. GER participated in the conceptualization of the grant applications and the overall study design, provided clinical expertise at all stages of the analysis, and assisted in framing the discussion. All authors read and approved the final manuscript.

## Pre-publication history

The pre-publication history for this paper can be accessed here:



## Figures and Tables

**Table 1:** Analytic Sample Means or Percentages.

	**Veterans**		**Non-Veterans**	
			
	**Over-Reporters**	**Accurate Reporters**	**Over-Reporters**	**Accurate Reporters**
	
***Covariates***				
Age (years)***	74.57	74.86	80.64	79.11
Race				
White (reference group)	90.1	92.0	74.1	79.8
Hispanic**	2.7	1.7	14.7	8.2
African American*	7.2	5.5	9.9	10.7
Other Race	0.0	0.8	1.3	1.3
High School Education***	64.1	67.5	35.0	45.1
HH Income < $15,000***	25.6	17.3	60.2	37.3
Total Wealth <= $19,000***	17.4	9.8	24.9	21.0
Self-Rated Health:				
Excellent**	4.7	15.1	5.2	11.5
Very Good**	10.9	26.6	15.0	20.9
Good (reference group)	30.5	31.8	32.3	33.4
Fair	28.1	19.9	21.4	23.7
Poor***	25.8	6.6	26.1	10.5
# ADLs w/difficulty***	0.38	0.14	0.56	0.26
# IADLs w/difficulty***	0.39	0.24	0.76	0.44
Cancer***	18.7	14.8	28.4	11.0
Diabetes	14.2	12.9	20.4	9.5
Heart Disease***	53.0	30.2	45.5	31.7
Lung Disease	19.0	10.7	13.4	11.5
Stroke	15.1	8.9	10.9	10.1
Cognitive Status (TICS-7)***	12.44	12.92	10.64	11.56
CES-D Symptoms***	2.14	1.12	1.98	1.58
				
***Selection Bias Adjustment***				
Propensity Score	0.63	0.63	0.64	0.64
				
***Intervening Variable***				
Any ACSC Admission**	21.3	11.2	25.6	14.5
				
**Weighted N**	96	792	60	574

Note: Weighted analyses adjust for the unequal probabilities of selection due either to the multi-stage cluster sampling design and/or the over-sampling.

*p < .05 **p < .01 ***p < .001

**Table 2:** Adjusted Hazards Ratios (AHRs) from the Mortality Models.

	AHRs	(95% C.I.)
***The Four Comparison Groups***		
Veterans Who Over-Reported (Dual Use of VHA & Medicare)	1.561**	(1.120, 2.177)
Veterans Who Accurately Reported	1.168	(0.982, 1.389)
Non-Veterans Who Over-Reported	0.775	(0.529, 1.136)
Non-Veterans Who Accurately Reported (Reference Group)	1.000	
		
***Covariates***		
Age	1.099***	(1.083, 1.115)
Race		
White (reference group)	1.000	
Hispanic	0.868	(0.587, 1.284)
African American	0.809	(0.609, 1.074)
Other Race	0.843	(0.445, 1.598)
High School Education	1.033	(0.867, 1.231)
HH Income < $15,000	0.872	(0.722, 1.052)
Total Wealth <= $19,000	1.061	(0.857, 1.313)
Self-Rated Health:		
Excellent	0.757	(0.571, 1.004)
Very Good	0.752*	(0.604, 0.936)
Good (reference group)	1.000	
Fair	1.162	(0.958, 1.408)
Poor	1.574***	(1.210, 2.049)
ADLs	0.946	(0.849, 1.055)
IADLs	1.110*	(1.017, 1.211)
Cancer	1.203	(0.983, 1.473)
Diabetes	1.311*	(1.054, 1.630)
Heart Disease	1.197*	(1.023, 1.401)
Lung Disease	1.458***	(1.179, 1.802)
Stroke	1.439**	(1.154, 1.794)
Cognitive Status (TICS-7)	0.945***	(0.920, 0.971)
CES-D Symptoms	1.039	(0.995, 1.084)
Hospitalized	1.108	(0.893, 1.374)
		
***Selection Bias Adjustment***		
Propensity Score	0.893	(0.381, 2.093)
		
***Intervening Variable***		
Any ACSC Admission	1.874***	(1.552, 2.262)

Note: Weighted analyses adjust for the unequal probabilities of selection due either to the multi-stage cluster sampling design and/or the over-sampling.

*p < .05 **p < .01 ***p < .001
